# Effect of Diet Neutral Detergent Fibre Content on Dry Matter Intake, Nutrient Digestibility, Growth Efficiency and Carcass Characteristics of Finishing South African Mutton Merino Wether Lambs

**DOI:** 10.1002/vms3.70853

**Published:** 2026-02-12

**Authors:** Inalene De Klerk, Abraham Vlok Ferreira, Michael Denis Fair, Arnold Hugo, Ockert Bernard Einkamerer

**Affiliations:** ^1^ Department of Animal Science University of the Free State Bloemfontein Republic of South Africa; ^2^ Technical Executive Molatek Malelane Republic of South Africa

**Keywords:** digestion, dressing percentage, lucerne hay, maize meal, performance

## Abstract

A study was conducted to investigate the effect of incrementally decreasing the diet neutral detergent fibre (NDF) content on dry matter (DM) intake, nutrient digestibility, growth efficiency and carcass characteristics of finishing South African Mutton Merino wether lambs. Five isonitrogenous dietary treatments were formulated, differing in fibre and NDF contents, with the NDF content in descending order. Treatments contained 379 (CON), 314 (NDF1), 251 (NDF2), 192 (NDF3) and 143 (NDF4) gram NDF/kg DM, respectively. The digestibility study was conducted over a period of 7 days, whereas the production study was conducted for 61 days. Diet DM, organic matter (OM), non‐structural carbohydrate (NSC), CP, NDF, acid detergent fibre (ADF) digestibility, as well as metabolisable energy (ME) content, were highest in diets with the lowest NDF content (NDF4 treatment). Diet DM intake was however the lowest for treatment NDF4. An increase in lamb feed conversion ratio (FCR: all treatments compared to CON) and ME utilised/kg live weight gain (NDF2 and NDF3 compared to CON: lower values) were also evident for increasing treatment NDF content. A low dietary NDF content also positively affected most of the carcass characteristics. The advantage of feeding a highly digestible and low‐NDF diet seems to have had the desired effect on the growth efficiency and carcass quality of South African Merino wether lambs. The observed increase in fibre (ADF and NDF) digestibility as NDF (lucerne hay) inclusion decreased, contrasts the reports of previously concluded studies and requires further investigation.

## Graphical abstract

Feeding South African Mutton Merino lambs low‐NDF lucerne hay diets improved nutrient digestibility, energy utilisation, growth efficiency, and carcass quality. Despite lower intake at the lowest NDF level, fibre digestibility and feed conversion improved, indicating that highly digestible, low‐NDF finishing diets enhance lamb growth and carcass traits.

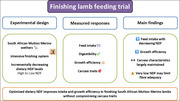

## 1 Introduction

Sheep breeds all vary in their growth and maturity characteristics. The South African commercial sheep industry is made up of a spectrum of breeds that are classed as wool type, dual‐purpose, meat type, and indigenous fat‐tail (Van Der Merwe and Brand 2020). One of the most popular breeds in commercial production systems in South Africa is the South African Mutton Merino (Van Der Merwe and Brand 2020). This breed was not initially developed in South Africa; however, it has undergone such a metamorphosis that it no longer bears much, if any, resemblance to its European ancestor (German Merino). It is a dual‐purpose (mutton and wool) sheep breed and is marketed at weaning either to a feedlot or directly to a slaughterhouse (Neser et al. 2000). This breed was developed through selection for improved wool quality as well as carcass conformation, with the main characteristic of a high growth rate (Skele et al. 2024).

The classic function of the gut is the digestion of feed into small molecules by means of enzymes and microbial fermentation, which can then be absorbed into the body to be utilised. Diet composition (ingredients, nutrients and additives) can influence the development and function of the digestive system, including the microbiota composition (Celi et al. 2017). Livestock feeds contain appreciable amounts of carbohydrates (Kellems and Church 2010) as their main energy source. The network formed by fibres within the cell wall (cellulose–lignin–hemicellulose) is believed to define its functional properties (Ana et al. 2022). Plant cell walls, which is measure as fibre (Oh et al. 2016), can account for up to 25% to 80% of forage dry matter (DM) (NRC 2007). Dietary NDF plays a crucial role for ruminant nutrition, serving as the most reliable indicator for cellulose expression (Shi et al. 2023) and an important parameter of forage quality (Oba and Allen 1999). It is also more closely associated with voluntary intake (Mertens 1992; Allen 2000) and gastrointestinal fill than any other measure of fibre (Van Soest et al. 1991). The fibre content of ruminant diets is also inversely related to its net energy (NE) content (De Klerk et al. 2025). Nutrient digestibility is typically related to the maturity of the fibre source (Macdonald et al. 2021). Decreasing dietary fibre content for example decreases DM and organic matter (OM) digestibility linearly (Lascano and Heinrichs 2011).

Recently, the South African Mutton Merino was compared to six other sheep breeds (Dohne Merino, Dormer, Dorper, Meatmaster, Merino, and Namaqua Afrikaner—ewes and rams) in South Africa to evaluate feedlot production characteristics between them (Van Der Merwe and Brand 2020). One standard diet was however provided to all the breeds. The quantity of dietary fibre affecting nutrient digestibility and animal performance were also extensively researched. More recent research (Mirzaei‐Alamouti et al. 2021) stated that there is ample research literature on energy and protein requirements within modern feeding models for sheep. Still, none of the existing feeding standards or models reports optimal dietary fibre (NDF) concentrations for sheep (Mirzaei‐Alamouti et al. 2021). The specific effect of NDF content within diets on lamb growth efficiency as well as small ruminant carcass characteristics, with special reference to the South African Mutton Merino breed, requires attention. Hence, using NDF as a collective term representing dietary treatments in the current study was unequivocal. Limited research was conducted on the South African Mutton Merino, specifically to what extent a decreasing diet NDF content affects its growth efficiency within an intensive finishing environment. Also, how this breed reacts when fed a decreasing dietary NDF content—increased concentrate diet—has not been evaluated in the past. The current study was therefore conducted to address the lack of breed‐specific information on optimal dietary NDF concentrations for South African Mutton Merino lambs by evaluating the effects of incrementally decreasing NDF levels on voluntary intake, nutrient digestibility, growth efficiency, and carcass characteristics under intensive finishing conditions.

## 2 Materials and Methods

This study was conducted at the Paradys experimental farm of the University of the Free State. The experimental farm is situated approximately 18 km south of Bloemfontein, along the Reddersburg road in the Free State province, South Africa, with an altitude of 1424 m above sea level. The exact coordinates are 29°06‘27.1“ latitude and 26°11’10.9” longitude. All procedures conducted during this study were approved by the Interfaculty Animal Ethics Committee for Animal Experimentation at the University of the Free State (Animal Experiment No. 15/2014).

### 2.1 Experimental Animals, Trial Design and Housing

The production study consisted of 50 South African Mutton Merino wether lambs (29.14 ± 1.66 kg; mean ± SD) and was conducted over a period of 61 days. The lambs were randomly assigned to five dietary treatments, culminating into a randomised trial design, with 10 lambs per treatment. Each lamb represented an experimental unit. Thirty lambs (45.65 ± 2.58 kg; mean ± SD) were randomly allocated to the same dietary treatments within the digestibility study, with 6 lambs per treatment representing the same trial design.

All animals were castrated with an elastic band at 8 weeks of age on the farm of origin, weaned 4 weeks prior to entering the trial and subjected to a standard health and vaccination program 3 weeks before the study started. The animals were injected with an antiparasitic remedy, dosed for tapeworm and inoculated against pulpy kidney, malignant oedema, blackquarter, tetanus, pasteurellosis as well as pneumonic and septicaemic pasteurellosis.

The lambs were housed individually in pens (1.404 m^2^) on elevated wooden slatted floors within a naturally ventilated building and the pens cleaned twice a week to ensure and maintain a good hygienic environment.

### 2.2 Experimental Diets and Trial Procedures

Five isonitrogenous experimental diets were formulated to facilitate an incremental decrease in the NDF content. Diet formulation followed NRC (2007) recommendations for late‐maturing 40 kg lambs growing at ∼300 g/day, with an expected intake of 1.54 kg DM/day, calculated at 3.84% of body weight. The dietary ingredients chosen are commonly used in sheep finishing diets in South Africa. Lucerne hay was used as the fibre source. As lucerne hay inclusion decreased, a concomitant increase in maize meal inclusion followed. Treatments were therefore defined according to NDF content in descending order and described as CON (379 g/kg NDF), NDF1 (314 g/kg NDF), NDF2 (251 g/kg NDF), NDF3 (192 g/kg NDF) and NDF4 (143 g/kg NDF), respectively (Table 1). Maize cobs were included in treatments CON and NDF1 to formulate for NDF content without affecting diet crude protein (CP) content.

**TABLE 1:** The physical and chemical composition of the five experimental diets formulated according to incrementally decreasing NDF content.

	Treatment diets^1^				
	CON	NDF1	NDF2	NDF3	NDF4
**Ingredients composition, g/kg**					
Maize meal	217	324	434	538	644
Molasses syrup	5.1	−	5.9	42	80
Soy oil	23	25	25	28	30
Maize germ oil	18	13	9	5	−
Lucerne hay	674	593	450	300	150
Maize cobs	42	8	−	−	−
Soybean meal	−	19	55	60	60
Urea	1.5	0.8	−	4.5	9.0
Limestone	−	−	3	6	10
Monocalcium phosphate	2.7	1.0	0.6	0.5	−
Ammonium chloride	10	10	10	10	10
Salt	5	5	5	5	5
Premix	2.5	2.5	2.5	2.5	2.5
**Chemical composition, g/kg DM**					
Dry matter	916	959	909	903	897
Organic matter	896	895	913	924	940
Crude protein	155	162	164	168	152
Non‐structural carbohydrate^2^	312	368	442	507	591
**Neutral detergent fibre**	**379**	**314**	**251**	**192**	**143**
Acid detergent fibre	256	216	161	116	75
Ether extract	52	56	51	53	51
Ash	104	105	87	76	60
Calcium	11.0	9.6	8.8	8.2	7.8
Phosphorus	3.4	3.1	3.0	2.8	2.5

^1^ Treatment diets: Neutral detergent fibre (NDF) content of diet (DM)—CON = 379 g/kg, NDF1 = 314 g/kg, NDF2 = 251 g/kg, NDF3 = 192 g/kg, NDF4 = 143 g/kg.

^2^ Non‐structural carbohydrate content calculated according to Van Soest et al. (1991).

All treatment diets were fed in a ground mash form, and no feed was processed after mixing. Lucerne hay was milled through a 20 mm sieve. No additives nor rumen modifiers were included in the diets.

At the onset of the production study (day 0) the lambs were subjected to a 7‐day adaptation period. Lucerne hay was provided on an ad libitum basis where each respective treatment diet was increased incrementally (100 g/day). After dietary adaptation, the additional lucerne hay was removed and the animals were fed the respective experimental diets on an ad libitum basis for the remainder of the experiment (total of 54 days). The animals were fed twice daily, at 08h00 and at 16h00. Feed intake was recorded on a weekly basis. Fresh, clean water was freely available to all the animals.

The digestibility study was conducted over a period of 7 days after a 7‐day adaptation whereas the lambs were offered the same experimental diets fed during the production study. A sequential method of feed allocation was followed to avoid variation in assessing the voluntary feed intake of sheep. Each animal was provided a 15% refusal level of intake. The lambs in the digestibility study were fed twice daily (08h00 and 16h00). Feed refusals were collected every morning before the lambs were fed.

### 2.3 Chemical Analysis

All samples were analysed for DM according to the Association of Official Analytical Chemists (AOAC) method 934.01 for chemical procedures (AOAC 1990). The DM content of the feed was determined in the physical form which the feed was presented to the lambs. The CP (AOAC method 990.03 – nitrogen content × 6.25), ash (AOAC method 942.05) and OM were also analysed according to AOAC (1990), whereas the NSC content was determined by the equation as described by Van Soest et al. (1991). The NDF (Procedure A) analytical fraction was determined according to the method of Van Soest et al. (1991) using the ANCOM^200/220^ Fibre Analyser (ANCOM Technology Corp., Fairport, NY, USA). Total ADF (AOAC method 973.18) was analysed according to AOAC (1990). Total lipid (ether extract—EE) was extracted according to the AOAC method 920.39 (AOAC 1990). Gross energy was determined using a Leco AC500 Isoperibol Calorimeter (Leco Corp., St. Joseph, MI) following the American Society for Testing and Materials (ASTM 2009) standard D5865 (Cantrell et al. 2010). Metabolisable energy (ME) was calculated from digestible energy values by multiplying the digestible energy by a factor of 0.81 (McDonald et al. 2011) to compensate for energy losses in the urine and methane gas. Energy losses from urine and combustible gasses are highly predictable, and therefore digestible energy and ME are highly correlated (NRC 2007).

### 2.4 Carcass Evaluation

All lambs were slaughtered on day 61 at a commercial abattoir to record cold carcass weight 24 h after refrigeration at 2°C (Fisher and de Boer 1994). The dressing percentage was calculated from the cold carcass weight. The external length, shoulder circumference and buttock circumference of each carcass were also recorded.

Meat evaluation was performed on the left side of each carcass between the 12th and 13th rib (thoracic vertebra) and fat depth measured with a calliper (Electronic digital calliper; Omni‐Tech) 45 and 110 mm from the mid dorsal line (Carson et al. 1999). The area of the longissimus muscle (*Musculus longissimus dorsi*) was traced directly onto transparent film between the 12th and 13th rib (Edwards et al. 1989). The traced outline was scanned with a scale bar and the eye muscle area measured using a video image analysis system (Soft Imaging System: analysis 3.0). The video image analysing system was calibrated with the scale bar.

### 2.5 Statistical Analysis

The data was analysed as a completely randomised design using the General Linear Model (GLM) procedures of the Statistical Analysis System (SAS) program [Statistical Analysis Software (SAS) 1999]. Means were compared using the LSMEANS/DIFF with treatment as fixed effects. For post hoc analysis, Tukey's honest significant difference (HSD) test was used to identify significant differences between treatment means and significance was declared at the 5% probability level.

The description of the model used for ANOVA analysis is shown:

Yij=μ+ti+εij,

where *Yij* is the individual observations (dependent variable) of the *i*th treatment (independent variable) and the *j*th random error, *μ* is the general effect, *ti* is the effect of the *i*th treatment, *εij* is the random variation or experimental error.

The *i*th treatment effect (dietary NDF content) during this study was defined as:

$$\begin{array}{ccc} i_{1} & = & 379\;g/\mathrm{kg}\;\mathrm{NDF}\;\left(\mathrm{CON}\right),i_{2}=314\;g/\mathrm{kg}\;\mathrm{NDF}\;\left(\mathrm{NDF}1\right),i_{3}\\ & = & 251\;g/\mathrm{kg}\;\mathrm{NDF}\;\left(\mathrm{NDF}2\right),i_{4}=192\;g/\mathrm{kg}\;\mathrm{NDF}\;\left(\mathrm{NDF}3\right),i_{5}\\ & = & 143\;g/\mathrm{kg}\;\mathrm{NDF}\;\left(\mathrm{NDF}4\right),\;\mathrm{presented}\;\mathrm{on}\;a\;\mathrm{DM}\;\mathrm{basis}. \end{array}$$

The data set was also analysed using analysis of covariance (ANCOVA) of the SAS program to test the possible effect of weaning weight on wether lamb performance response. The description of the model used for ANCOVA analysis was:

$$Yij=\mu +ti+\gamma \left(Xijk\right)+\varepsilon ij,$$

where *Yij* is the individual observations (dependent variable) of the *i*th treatment (independent variable) and the *j*th random error, *μ* is the general effect, *ti* is the effect of the *i*th treatment, *γ*(*Xijk*) is the covariate term for starting weight, εij is the random variation or experimental error. Tukey's HSD test was used to identify significant differences between treatments and significance was also declared at the 5% probability level. Start weight (weaning weight) as covariate was deemed significant for feed conversion ratio (FCR); MJ ME used per kg live weight gain, cold carcass mass, shoulder circumference, buttock circumference as well as fat thickness measured 45 mm from the mid dorsal line. The adjusted means and standard errors (SE) were used as presented within the ANCOVA model only.

## 3 Results and Discussion

### 3.1 Dry Matter Intake and Nutrient Digestibility

Dry matter intake of NDF4 was lower compared to that of NFF1, NDF2 and NDF3 (*p* < 0.05) (Table 2). This is in contrast to most literature as diets with excessive fibre from roughage has proven to rather decrease feed consumption (Mirzaei‐Alamouti et al. 2021). The length of feeding could however affect these results and the minimum necessary to establish stable intake for sheep was documented to be between 12 and 15 days (Blaxter et al. 1961), whereas it was seven consecutive days in the current study. The intake of sheep kept in metabolism pens are less variable (Minson 1990). Feed intake is also less variable for concentrate‐fed than forage‐fed wethers (Ellison et al. 2022). Feed duration could seem like a limitation of the digestibility study for accurate DM intake evaluation. Dry matter intake within the production data presented by De Klerk et al. (2025) was also significantly lower for treatment NDF4, compared to treatments NDF1 and NDF2. The animals in the production study were fed for 61 days. According to Allen (2000), changing the NDF content of a lactating dairy cow diet by substituting grain for forage will increase DM intake until it is no longer limited by gut fill and will eventually decrease when limited by an excess of metabolic fuels. An increase in diet NDF content is associated with a linear reduction in DM intake (Shi et al. 2023).

**TABLE 2:** The effect of decreasing NDF content in South African Mutton Merino wether lamb diets on DMI, apparent digestibility and ME content [mean (SE)].

	Treatment diets^1^					Significance	
Parameter	CON	NDF1	NDF2	NDF3	NDF4	*p*‐value	SEM
Dry matter intake, g/sheep/day	1455^ab^ (89.5)	1617^a^ (92.5)	1552^a^ (59.3)	1512^a^ (36.0)	1193^b^ (84.7)	0.0059	41.507
**Digestibility coefficient**							
Dry matter	0.66^d^ (0.011)	0.73^c^ (0.019)	0.77^bc^ (0.011)	0.82^ab^ (0.006)	0.86^a^ (0.016)	<0.0001	0.014
Organic matter	0.70^d^ (0.011)	0.76^c^ (0.018)	0.79^bc^ (0.011)	0.83^ab^ (0.006)	0.87^a^ (0.016)	<0.0001	0.012
Non‐structural carbohydrate	0.84^b^ (0.025)	0.91^ab^ (0.020)	0.91^ab^ (0.012)	0.93^a^ (0.008)	0.94^a^ (0.019)	0.0058	0.010
Crude protein	0.70^c^ (0.013)	0.74^bc^ (0.018)	0.75^b^ (0.010)	0.80^ab^ (0.009)	0.82^a^ (0.018)	<0.0001	0.010
Neutral detergent fibre	0.57^b^ (0.013)	0.58^b^ (0.022)	0.58^ab^ (0.017)	0.60^ab^ (0.015)	0.65^a^ (0.017)	0.0251	0.009
Acid detergent fibre	0.54^b^ (0.013)	0.55^b^ (0.024)	0.54^b^ (0.017)	0.57^ab^ (0.019)	0.67^a^ (0.045)	0.0061	0.014
Ash	0.32^c^ (0.029)	0.46^b^ (0.034)	0.50^b^ (0.045)	0.58^ab^ (0.015)	0.67^a^ (0.018)	<0.0001	0.025
Ether extract	0.86^b^ (0.009)	0.85^b^ (0.016)	0.87^ab^ (0.010)	0.90^ab^ (0.007)	0.91^a^ (0.011)	0.0032	0.006
Metabolisable energy, MJ/kg DM	9.06^c^ (0.125)	9.75^bc^ (0.285)	10.25^b^ (0.162)	11.09^a^ (0.086)	11.45^a^ (0.234)	<0.0001	0.180

^a,b,c,d^Mean values with different superscripts in the same row differ significantly (*p* < 0.05).

^1^ Treatment diets: Neutral detergent fibre (NDF) content of diet (DM)—CON = 379 g/kg, NDF1 = 314 g/kg, NDF2 = 251 g/kg, NDF3 = 192 g/kg, NDF4 = 143 g/kg.

Abbreviation: SEM, standard error of the mean.

Neutral detergent fibre content [cell wall components: Kawas et al. (1991)] of the diet is more related to intake (Mertens 1992; Allen 2000) and gastro‐intestinal fill than any other measure of fibre (Van Soest et al. 1991), even more than ADF (Jung and Allen 1995). The filling effect of a ration is determined by the initial bulk density of a feed, as well as its filling effect in the rumen. Forage NDF is less dense initially, digests more slowly, and is retained in the rumen longer than concentrates (Allen 2023). A signal from ruminal distension by undigested feed residues begins to dominate the control of voluntary feed intake (Allen 2023), which then declines. The main issue is a higher amount of undigested cell wall constituents (Aregheore 2000). This is an important factor which could limit intake and normally occurs with low quality roughages (Pathak 2008). In contrast, it has been proven however that potentially digestible NDF (pdNDF) has a slower passage rate than indigestible NDF (iNDF) and that passage of small particles out of the rumen is the rate‐limiting step for the disappearance of particles via passage (Krämer et al. 2013). Therefore, as feed (grass silage) digestibility decreases rumen outflow rate can increase and the pool sizes of DM, NDF, and iNDF increase (Kuoppala et al. 2009). This was the case in the present study as it seems that DM, fibre and nutrient digestibility did play an important role on the voluntary feed intake of South African Mutton Merino lambs. It has to be noted however that the relationship between NDF and voluntary intake is complex (Mertens 1992). This is purely because not all NDF acts alike when consumed due to its density (physical form), chewing requirement, digestion rate and extent of digestion that varies among sources (Mertens 1992). It is known that the responses of lambs to dietary NDF depend on the type, amount, and quality of the fibre source used, considering that fibre quality affects rumen fill, and that sheep have a higher ruminal passage rate than cattle and can ruminate very fine particles that would escape rumination in cattle (Mirzaei‐Alamouti et al. 2021).

The energy required for productive purposes as well as the satiety of nutrients consumed are also important to consider as factors regulating feed intake (Blaxter et al. 1961). Feed intake of lambs fed a high concentrate diet is therefore controlled by the energy demand of the animal (Haddad and Husein 2004). It is therefore doubtful that only rumen distention or gastro‐intestinal tract fill limits voluntary intake of high‐energy pelleted diets. A chemostatic or thermostatic mechanism probably regulates the voluntary consumption of rations of this type, where it appears that acetate and ketones would be the most probable signal compounds for a chemoreceptor (Simkins et al. 1965). Dry matter intake of ruminants is therefore regulated by both bulk density and physical fill of the digestive tract (Allen 2023). It seems that the voluntary feed intake of South African Mutton Merino's in the current study has probably been affected by a chemostatic effect rather than physical fill when lucerne hay was used as roughage source to formulate for (NDF4—143 g/kg DM).

Insufficient NDF in a diet (i.e. containing high concentrate) leads to a significant reduction in rumination time, saliva secretion, with a decrease in pH value (Shi et al. 2023) This is associated with metabolic disorders such as acidosis which may reduce appetite and depress intake (Mao and Wang 2025). This could also explain the decreased DM intake of lambs in the current study. The minimum adequate NDF content to maintain rumen health for sheep were proposed to be between 15% and 20% NDF when utilising *Medicago sativa* (lucerne) hay milled through a 12.5 mm screen (Smith 2008). The NDF content of NDF4 (143 g/kg DM) was however below this recommended NDF value. Van Soest et al. (1991) cautioned that any system that sets fixed values for dietary fibre requirements for ruminants is inadequate due to the fact that rumen size and level of intake and production affect that requirement. It was proposed that small ruminants adapt their feeding behaviour to the fibre proportion of the feed offered, other than where a reduction in fibre concentration below 20% has been shown to increase the risk of ruminal acidosis in feedlot cattle, which can challenge animal health and welfare (Mirzaei‐Alamouti et al. 2021).

Fimbres et al. (2002) found that the DM intake of male Pelibuey lambs within a digestibility study increased linearly following increased hay inclusion (decreasing sorghum grain by 10%, 20% and 30%, respectively) which the authors clarified was a result of the animals increasing their DM intake due to the dilution of energy density of the diets. These authors acknowledged that this was not in agreement with most literature. In contrast, the DM intake of high concentrate (15:85; roughage:concentrate ratio) versus high fibre (60:40; roughage:concentrate ratio) diets fed to Awassi lambs was however similar (*p* > 0.05) (Haddad and Husein 2004). The roughage:concentrate ratio for the CON treatment was 72:28 and that of the NDF4 treatment 15:85.

Lipid levels exceeding 5% (Seabrook et al. 2011) to 7% of the diet may result in decreased fibre digestibility by inhibiting fibrolytic bacteria (Palmquist, 1988) and reduce feed intake (Seabrook et al. 2011; Fiorentini et al. 2015). However, the lipid content was comparable between the diets in the current study and was on average 52.6 g/kg DM (Table 1).

Both DM and OM digestibility were higher for treatments NDF3 and NDF4 compared to treatments CON and NDF1. Cereal grains are rich in energy (Humer and Zebeli 2017) and support their high digestibility as seen with the increased maize content in the current diets (Table 1). Dry matter digestibility increased with a decrease in hay level of lamb finishing diets in research conducted by Fimbres et al. (2002) and is in comparison with the results of the current study. Fimbres et al. (2002) explained that the increase in DM digestibility was probably due to a decreased rate of passage (Fimbres et al. 2002). This was also discussed earlier (Kuoppala et al. 2009). Diet digestibility is however affected by voluntary feed intake (NRC 2001) and the extent of the depression in digestive efficiency caused by increased intake is related to the proportion of grain in the diet. At equal DM intake, DM digestibility was proven to be significantly higher when concentrate diets are fed (60% maize and 40% hay) compared to only hay (Dehority 2003). This is comparable in the current study as DM digestibility was higher for NDF4 (0.86: with 644 g maize/kg feed) compared to the CON treatment (0.66: with 217 g maize/kg feed) (Table 2). Dehority (2003) explained that when feed consumption is constant, the rumen can even contain undigested residues of highly digestible feed for an extended period of time. Dry matter and OM apparent digestibility for lambs fed a high concentrate diet were also higher compared with lambs fed a high fibre diet as evaluated by Haddad and Husein (2004). Kawas et al. (1991) similarly found that the total DM digestibility of dairy goat diets increased following an increased proportion of concentrate feeding.

Research conducted by Fimbres et al. (2002) proved that nitrogen digestibility of lamb finishing diets increased with a decrease in hay level of the diet. The higher nitrogen digestibility was acknowledged due to an increased availability of energy content of the diets containing higher levels of grain sorghum (Fimbres et al. 2002). Haddad and Husein (2004) also found an increased protein digestibility within lambs fed a high concentrate diet (85:15 concentrate: roughage ratio; comparable to treatment NDF4 in the current study). This is in accordance with the current study and clearly depicted regarding the significant higher CP digestibility of treatment NDF4 compared to treatments CON and NDF1, as well as treatment NDF3 compared to treatment CON (Table 2). The ammonia‐nitrogen concentration within continuous culture fermenters was higher with a high (70:30) versus a low (30:70) forage diet (Gudla et al. 2012). Gudla et al. (2012) explained that the lower ammonia‐N concentration as a result of the low forage diets may be due to lower protein digestibility under low pH conditions, and/or the lower protein degradability in forage relative to concentrates. The ammonia‐N was not determined in the current study. Protein digestibility of feedstuffs classified as roughages is generally lower (Kellems and Church 2010).

Neutral detergent fibre (NDF4 compared to treatments CON and NDF1) and ADF (NDF4 compared to treatments CON, NDF1 and NDF2) digestibility were higher with a low NDF content within the finishing diet for the finishing lambs in the current study. This is in contrast to most literature. As there is a positive relationship between voluntary forage intake and total NDF digestibility (Detmann et al. 2024). Acid detergent fibre is again generally better correlated with diet digestibility than NDF (Van Soest 1994) and therefore the susceptibility of fibre digestion by microorganisms due to the chemical structure of plants is paralleled to its ADF and lignocellulose content (Dryden 2008). Acid detergent lignin, as the main component of Klason lignin, most negatively affects digestibility of NDF in forage samples dominated by lucerne and maize (Huhtanen and Krizsan 2023). The degree of protection against digestion conferred by lignin is however not linearly related to its lignin content. Differences in cellulose digestibility have been reported between forages with identical lignin contents (Dryden 2008).

The concentration of dietary NDF has a significant positive correlation with rumen pH. Insufficient NDF content in the diet leads to a significant reduction in rumination time, chewing time, and saliva secretion, resulting in a decrease in pH value (Shi et al. 2023). Rumen pH is maintained by the bicarbonate, phosphate, and protein buffering system contained in saliva (Castillo‐Lopez et al. 2021). Most rumen microbes are sensitive to low rumen pH conditions (Jiyana et al. 2021), and the competition for nitrogen between non‐fibrous carbohydrate (NFC) and fibre digesting microbes may also be the reason for the decline in efficiency of NDF digestion when higher levels of NFC are fed (Heldt et al. 1999).

Even though fibre digestibility and intake are affected by dietary starch content (Dove 2002), two further arguments have to be stressed. First, the reduction in whole‐tract digestibility may not be as great as the reduction in cell‐wall digestibility in the rumen, indicating that there are compensating increases in digestibility elsewhere in the tract. Secondly, the degree of substitution occurring with high‐starch supplements is frequently larger than the extent of depression in cell‐wall digestibility, indicating that other factors are involved in causing the substitution between roughage and supplement (Dixon and Stockdale 1999).

In contrast to most literature, and in comparison to the results of the current study, Fimbres et al. (2002) found however that NDF digestibility increased (from 0% to 30%) with a decrease in hay level of the diet fed to finishing lambs. It was proven by Ludden et al. (1995) that even when diet DM digestibility decreased as a percentage of intake following increasing soybean hull inclusion (comparable to the current study), NDF digestibility was however not affected. The positive effect on NDF and ADF digestibility as diet NDF content decreased within the current study is not commonly reported in literature. This could be related to lucerne hay as the main roughage source and requires further investigation. However, Lascano and Heinrichs (2011) also recorded a higher NDF and ADF digestibility when the concentrate level increased (decreased corn stover) in dairy heifer diets. The authors discussed that it could possibly be related to digesta retention time (decreased with low‐concentrate diet) and roughage source (corn stover and soybean hulls). The latter specifically related to its cellulose content and digestibility (higher for soybean hulls) (Lascano and Heinrichs 2011). This reiterates that fibre digestibility is affected by feed intake, that is, digesta passage rate, and fibre composition, again related to fibre source.

Non‐structural carbohydrate digestibility was higher for treatments NDF3 and NDF4 compared to the control treatment (CON) in the current study. This result was expected. Cereal grains have long been an important component of the diet in ruminants because they are high in starch which enables high energy density diets (Humer and Zebeli 2017) and proof that it is highly digestible. Amylolytic bacteria is primarily responsible to ferment starch (Reddy et al. 2008) and their rumen concentration could have been favoured with the increased maize inclusion. In comparison, Fimbres et al. (2002) also found that dietary NSC digestibility increased with a decrease in hay level (from 30% to 0%) of the diet fed to finishing lambs. Increased rumen starch degradation as a percentage of DM could however result in a significant depression in daily DM intake of ruminants. Greater ruminal fermentation increases the production of fermentation acids with a higher proportion of propionate which has greater hypophagic effects in ruminants (Oba and Allen 2003). This could however relate to the effect of dietary treatment on lamb intake discussed earlier.

Ether extract digestibility was also higher for treatment NDF4, compared to the CON and NDF1 treatments. It is unclear if NDF content influences lipid digestibility and more research is necessary in this regard. Dietary forage level does however affect the growth of *Anaerovibrio lipolytica* (Gudla et al. 2012) which decreases with high fermentable carbohydrate and low forage diets (Bach et al. 2023). It was however presented by Doreau and Ferlay (1994) that the fatty acid balance at the end of the rumen is often negative in ruminant diets supplemented with lipids. According to them the cause of this apparent disappearance of fatty acids is not known. Lipids are first hydrolysed largely in the rumen (hydrogenation of unsaturated fatty acids), which decreases when the level of concentrates increases in the diet. Digestibility of fatty acids in the small intestine of ruminants normally ranges from 70% to 90% (Doreau and Ferlay 1994).

The ME content of diets evaluated was higher for treatments NDF3 and NDF4 compared to the other treatments. This was similar to treatment effect on DM and OM digestibility. In comparison to the current study, Ludden et al. (1995) found that dietary gross energy digestibility increased as soybean hulls decreased (60% to 0%) and maize increased within the diets, causing a linear increase in the digestible energy concentration of the diet (66.7% to 80.5%, respectively) (Ludden et al. 1995). Increasing dietary roughage in feedlot diets decreases DM digestibility with a resultant reduction in diet energy content (Weiss et al. 2017) and is depicted in the lower diet ME content within the current study as affected by a high NDF content (CON, NDF1 and NDF2 compared to treatments NDF3 and NDF4). As the digestibility of a diet is affected by feed intake, a reduction in feed intake may increase the energy value of any given diet (NRC 2001). This is plausible for the current study. Factors that can influence the energy value of the digestible OM content of a diet are the proportions of organic acids, soluble carbohydrates, fibre and fats (Minson 1990). The total digestible nutrient content of a feed represents the total digestible OM content (NRC 2007). At a maintenance, level of nutrition approximately 7% to 9% of the gross energy of the food (11% to 13% of the DE) is lost as methane. At higher levels of feeding, the proportion falls to 6% to 7% of gross energy, the reduction being most marked for highly digestible feeds (McDonald et al. 2011).

Increasing diet fermentability can increase the efficiency of rumen fermentation leading to less enteric methane energy and heat losses (Nasrollahi et al. 2019). Heat increment of digestion and metabolism for carbohydrates are higher compared to fats for instance (Grummer 1992). The Cornell Net Carbohydrate and Protein System is a model that can predict rates of feedstuff degradation in the rumen, the passage of undegraded feed to the lower gut and the amount of ME (and protein) that is available to the animal. The carbohydrate content of feeds for ruminants is divided by this model into four fractions: sugar, starch and pectin, available fibre and unavailable fibre (Sniffen et al. 1992). The first three fractions mentioned can be fermented in the rumen at fast, moderate and a slow speed, respectively. The unavailable fibre fraction is not fermentable in the rumen (Dong and Zhao 2013). Dong and Zhao (2013) explained that the rumen fermentation of cellulose and hemicellulose mainly produces acetate [lower with high concentrate diets: Martin et al. (2002)] and butyrate accompanied with the formation of carbon dioxide and hydrogen which are used for methane production. Whereas fermentation of sugars and NSC mainly produces propionate accompanied with an uptake of hydrogen. Hence in high roughage type of diets, rumen acetic and butyric acid (Fimbres et al. 2002) concentration increases with a resultant decrease in propionic acid concentration (Gudla et al. 2012). Digestion of starch in the small intestine is again energetically favourable compared to the rumen due to fermentation losses (Cerrilla and Martínez 2003). It is presumed that the fermentation of the available fibre fraction would thus produce more methane than that of the sugar, starch and pectin fractions (Dong and Zhao 2013). Hence more energy could be lost as methane when high‐fibre diets are fed/digested, with a resultant decrease in diet ME content. In addition, methanogenesis can be reduced when the ratio of acetate:propionate is reduced due to increased soluble carbohydrate content within a diet (Lee et al. 2003). Methane produced was however not measured in the current study and therefore could not be deemed as a definitive effect.

### 3.2 Lamb Growth Efficiency and Carcass Characteristics

The effect of decreasing dietary NDF content on the growth efficiency of South African Mutton Merino wether lambs is presented in Table 3. The DM intake and daily growth (average daily gain and final weight) of these animals were reported by De Klerk et al. (2025). To summarise, voluntary DM intake was similarly affected by finishing diet NDF content within the production study (1423, 1530, 1452, 1419 and 1309 g DM intake/lamp/day for treatments CON, NDF1, NDF2, NDF3 and NDF4, respectively), compared to the digestibility study. Metabolisable energy intake of the lambs were however higher (*p* < 0.05) for all treatments compared to that of the CON treatment. This resulted in an increased lamb final live weight and average daily gain, which was comparable with ME intake (12.45, 14.34, 13.87, 14.64 and 14.02 MJ ME intake/lamp/day for treatments CON, NDF1, NDF2, NDF3 and NDF4, respectively) (De Klerk et al. 2025). It is however inevitable that increasing dietary energy by reducing forage can give rise to an increase in body weight gain (Broderick 2003). Feeding grains to ruminants enables the high energy density of their diets to support production (Humer and Zebeli 2017). The fibre content of a ruminant diet is inversely related to its energy content (De Klerk et al. 2025). In contrast, incidences of rumen disorders could result when feeding diets rich in degradable starch (Humer and Zebeli 2017) and an associated negative effect on animal performance (Bach et al. 2023). Ludden et al. (1995) proved that the less efficient utilisation of diets based on 0% soybean hulls may have occurred because of metabolic upsets. Huber and Kung (1981) explained that the efficiency can decrease when the readily fermentable carbohydrate supplementation level is greater than 70% of the diet, and may be because of a rapid rate of NSC degradation.

**TABLE 3:** The effect of decreasing NDF content in finishing diets on the growth efficiency and carcass characteristics of South African Mutton Merino wether lambs [mean (SE)].

	Treatment diets^1^					Significance	
Parameter	CON	NDF1	NDF2	NDF3	NDF4	*p*‐Value	SEM
**Growth efficiency**							
FCR	5.57^a^ (0.114)	4.93^b^ (0.114)	4.55^bc^ (0.114)	4.18^c^ (0.114)	4.24^c^ (0.114)	<0.0001	0.051
MJ MEI/kg live weight gain	48.86^a^ (1.055)	46.24^ab^ (1.055)	43.42^b^ (1.055)	43.25^b^ (01.055)	45.45^ab^ (1.055)	0.0028	0.472
**Carcass characteristics**							
Cold carcass weight, kg	19.99^b^ (0.350)	21.99^a^ (0.350)	23.38^a^ (0.350)	23.40^a^ (0.350)	22.65^a^ (0.350)	<0.0001	0.156
Dressing percentage, %	44.65^c^ (0.35)	45.60^bc^ (0.48)	48.01^a^ (0.61)	46.86^ab^ (0.52)	47.11^ab^ (0.52)	0.0002	0.274
Shoulder circumference, cm	75.75^b^ (0.437)	77.52^a^ (0.437)	78.27^a^ (0.437)	78.61^a^ (0.437)	78.25^a^ (0.437)	0.0002	0.195
Buttock circumference, cm	64.01^b^ (0.728)	66.47^ab^ (0.728)	66.87^ab^ (1.728)	66.50^ab^ (0.728)	67.70^a^ (0.728)	0.0128	0.325
Carcass length, cm	60.10 (0.504)	61.20 (0.490)	61.65 (0.633)	61.80 (0.291)	63.10 (2.766)	0.6090	0.580
*M. longissimus dorsi* width, mm	59.42 (1.461)	58.31 (1.496)	60.17 (1.120)	61.00 (1.467)	61.74 (0.936)	0.3979	0.589
*M. longissimus dorsi* depth, mm	28.57^c^ (0.855)	29.64^bc^ (0.861)	33.87^a^ (0.921)	31.65^abc^ (1.110)	32.24^ab^ (0.750)	0.0013	0.473
*M. longissimus dorsi* area, mm	1490^b^ (49.6)	1567^b^ (64.5)	1720^ab^ (69.6)	1770^ab^ (100.6)	1903^a^ (66.5)	0.0016	37.342
Fat thickness 45,^2^ mm	3.94 (0.395)	3.99 (0.395)	4.14 (0.395)	4.91 (0.395)	3.72 (0.395)	0.2779	0.177
Fat thickness 110,^2^ mm	8.64 (0.915)	9.28 (0.996)	9.41 (1.010)	10.25 (1.065)	10.16 (0.750)	0.7433	0.417

^a,b,c^ Mean values with different superscripts in the same row differ significantly (*p* < 0.05).

^1^ Treatment diets: Neutral detergent fibre (NDF) content of diet (DM)—CON = 379 g/kg, NDF1 = 314 g/kg, NDF2 = 251 g/kg, NDF3 = 192 g/kg, NDF4 = 143 g/kg.

^2^ Fat thickness measured 45 and 110 mm from the mid dorsal line between the 12th and 13th thoracic vertebra, respectively.

Abbreviations: FCR, feed conversion ratio (kg DM feed intake/kg weight gain); MEI, metabolisable energy intake; SEM, standard error of the mean.

The effect of decreasing dietary NDF content on growth efficiency and carcass characteristics of South African Mutton Merino wether lambs are presented in Table 3.

Decreasing finishing diet NDF content increased South African Mutton Merino final live weight and average daily gain (CON compared to the other treatments) (De Klerk et al. 2025). The same associated increased growth efficiency (indicating lower values) with decreasing diet NDF content was also evident in the current study. Feed conversion ratio increased (lower values) for treatments NDF2, NDF3 and NDF4, compared to that of treatments CON and NDF1. Treatments NDF2 and NDF3 were also more efficient (lower values) in ME energy used for growth compared to the CON treatment in the current study. In contrast with the reports of the current study, Fimbres et al. (2002) determined that even though the DM intake of lambs increased following forage inclusion (up to 30%), it did not result in increased weight gain and feed efficiency. The authors did however acknowledge that high grain diets do result in more efficient growth response. The lower ME content of a roughage source (maize stover) can be ascribed to the inferior digestibility of its carbohydrate (NDF and NSC) fractions (Macdonald et al. 2021), which will have a negative effect on diet energy content when included at high amounts. Reducing the NDF concentration of a high‐rumen undegradable protein ration from 270 to 220 g/kg DM resulted in a decrease in DM intake that compensates for the increased energy density of the ration, thereby maintaining growth rate and improving FCR (Mirzaei‐Alamouti et al. 2021).

In low roughage diets, rumen propionic acid concentration is higher compared to acetic and butyric acids (Fimbres et al. 2002). Propionate is one of the main sources of energy for ruminants and it is energetically more effective compared to other volatile fatty acids (Ishlak et al. 2015). Volatile fatty acids account for 50% to 70% of digestible energy intake of small ruminants (NRC 2007). Therefore, any process, which increases the quantity of propionic acid absorbed, leads to less enteric methane and heat losses (Nasrollahi et al. 2019) and increases growth (Liu et al. 2022). Feeding flaked maize within hay‐based diets increases the efficiency of utilisation of ME for fattening (*k_f_*) in direct proportion to the level of grain in the diet, with little difference in response between sheep and cattle. This improvement in ME efficiency for maintenance increases from 71% to 79%, and for fattening from 29% to 61% as the percentage of flaked maize in the diet increased from 0% to 100%. This is not only attributed to the mentioned fall in the proportion of acetic acid in the rumen, but also reduced heat of fermentation and a smaller quantity of energy used to chew the cud (Blaxter and Wainman 1964).

Variations in efficiencies of utilisation of ME have been observed within ruminants (Dryden 2008). NDF digestibility for example can be seen as one of the main indicators of available energy for supplying the maintenance and production requirements of the host animal (Detmann et al. 2024). In contrast to the current study, the effects of various intake levels of structural carbohydrates (lucerne hay) as measured by dietary ADF content (from 14.3% to 25.7%) during the first 4 weeks of lactating dairy goats were determined by Santini et al. (1991) to estimate quantitative requirements for structural carbohydrates for these animals. As alfalfa hay increased (37.0 to 81.5%) (ground maize decreased: 51% to 16%) in the diets to formulate for increased ADF content, a similar increase in NDF (39.0% to 47.0%) was noted and used to compare data within their study. There were however no differences in body weight or body weigh change, as well as milk production according to dietary ADF (and NDF) content (Santini et al. 1991).

Cold carcass weight and shoulder circumference (CON vs. the other treatments), dressing percentage (NDF2, NDF3 and NDF4 vs. CON), buttock circumference (NDF4 vs. CON), as well as *Musculus longissimus dorsi* depth (NDF2 and NDF4 vs. CON, as well as NDF2 vs. CON and NDF1) and area (NDF4 vs. CON and NDF1) were higher (*p* < 0.05) following decreased dietary NDF content. These results are associated with the same and comparable significant (*p* < 0.05) dietary effects on lamb ME intake and growth response (De Klerk et al. 2025).

Feeds high in energy level (grain) increase the fat content of cold carcasses (Kim et al. 2023). The amount of visible fat is however a strong visual cue and perceived as negative by the consumer (Skele et al. 2024). Cattle fed a higher energy diet (grain‐based) was heavier and fatter in comparison with those fed a predominantly forage‐based diet (Muir et al. 1998). Lambs fed a high concentrate diet (15% alfalfa hay and 85% concentrate) also had higher (*p* < 0.05) hot (16.3 kg) and cold (15.7 kg) carcass weights as well as dressing percentage (48.5) compared to lambs fed a high fibre diet (60% alfalfa hay and 40% concentrate, resulting in 12.8 kg, 12.3 kg and 43.5% hot and cold carcass weights, as well addressing percentage, respectively) (Haddad and Husein 2004). However, Cooke et al. (2004) referred to the fact that varying the concentrate level in the diet of beef animals does not always affect dressing percentage. In studies where different proportions of acetic, propionic, and butyric acid concentrations were evaluated, there is an increase in *k_f_* as the proportion of propionic acid increases (Blaxter 1962). The lack of treatment effect (*p* > 0.05) on the fat thickness measured 45 and 110 mm from the mid dorsal line within the current study was therefore not expected. This is difficult to explain due to the significant effect of treatment on growth performance of the lambs. The carcass characteristics, muscular growth and carcass fatness of young bulls were also not affected by a high concentrate compared to a low concentrate (roughage:concentrate ratio) diets as evaluated by Marino et al. (2006). Damascus male kids confined indoors and fed concentrate had similar slaughter and carcass traits as those allowed 8 h per day grazing. The level of concentrate in the diet can have minimal influence on fat content of goat meat as well (Ponnampalam et al. 2024).

## 4 Conclusion

The NDF content of South African Mutton Merino wether diets seems to affect diet digestibility, lamb growth efficiency and carcass characteristics. In general, diet DM and nutrient digestibility were positively affected by a low NDF finishing diet content, which includes NDF and ADF digestibility. The diets’ composition, associative feed affects, and animal breed need further investigation on this subject. The recommendation is thus endorsed that feeding a diet to finishing South African Mutton Merino lambs with the least amount of NDF to still maintain a normal functioning rumen has the desired influence on animal growth efficiency, carcass weight and dressing percentage. More research is required to assess the effect of the same treatments on rumen fermentation patterns and microbial composition. The decrease in diet DM intake and increase in fibre (ADF and NDF) digestibility as NDF (lucerne hay) inclusion decreased is in contrast to most literature and requires further investigation.

## Author Contributions

I.d.K. and O.B.E. conceived and designed the study. I.d.K. and O.B.E. performed the experiments and collected the data. I.d.K. wrote the original draft. M.D.F. analyzed the data. A.V.F., A.H. and O.B.E. critically revised the manuscript and provided supervision of the study. A.H. and O.B.E. contributed to the methodology and resources. All authors approved the final version.

## Funding

This study was funded by the Directorate Research and Development of the University of the Free State, Bloemfontein, South Africa to develop PhD students.

## Data Availability

The data that support the findings of this study are available from the corresponding author upon reasonable request.
